# Regular consumption of soft drinks is associated with type 2 diabetes incidence in Mexican adults: findings from a prospective cohort study

**DOI:** 10.1186/s12937-020-00642-9

**Published:** 2020-11-20

**Authors:** Leticia Torres-Ibarra, Berenice Rivera-Paredez, Rubí Hernández-López, Francisco Canto-Osorio, Luz María Sánchez-Romero, Nancy López-Olmedo, Romina González-Morales, Paula Ramírez, Jorge Salmerón, Tonatiuh Barrientos-Gutiérrez

**Affiliations:** 1 Center for Population Health Research, National Institute of Public Health, Cuernavaca, Morelos Mexico; 2 Research Center on Policies, Population and Health, Faculty of Medicine, National Autonomous University of Mexico, Mexico City, Mexico; 3 Epidemiological Research and Health Services Unit, Mexican Institute of Social Security, Cuernavaca, Morelos Mexico

**Keywords:** Soft drinks, Type 2 diabetes, Adults, Cohort, Mexico

## Abstract

**Background:**

Although high consumption of soft drinks has been associated with excess of type 2 diabetes risk, the strength of this association in the Mexican population, where a type 2 diabetes genetic susceptibility has been well established, has been scarcely studied. This study aimed to estimate the risk of type 2 diabetes due to soft drinks consumption in a cohort of Mexicans.

**Methods:**

We used data on 1445 participants from the Health Workers Cohort Study, a prospective cohort conducted in Cuernavaca, Mexico. Soft drinks consumption was assessed with a semi-quantitative 116-item food frequency questionnaire. Incident type 2 diabetes was defined as self-report of physician-diagnosed type 2 diabetes, fasting glucose > 126 mg/dl, or hypoglycemic medication at any examination. Hazard ratios (HRs) and 95% confidence intervals (CIs) were estimated using Cox proportional hazard models.

**Results:**

With a total of 9526.2 person-years of follow-up, 109 incident cases of type 2 diabetes were observed. Type 2 diabetes incidence rate was 7.6, 11.0, and 17.1 per 1000 person-years across levels of soft drinks consumption of < 1, 1–4, and ≥ 5 servings/week, respectively (*p* < 0.001 for trend). The intake of ≥5 soft drinks/week was significantly associated with an increased risk of type 2 diabetes (HR 1.9 95% CI:1.0–3.5) compared with consumption of < 1/week (p-trend = 0.040). The HR was attenuated by further adjustment for body mass index (HR 1.5 95%CI:0.8–2.8) and abdominal obesity (HR 1.6 95%CI:0.8–3.0).

**Conclusions:**

The consumption of soft drinks was associated with a higher risk of type 2 diabetes in a cohort of Mexican adults. Our results further support recommendations to limit soft drinks intake to address the growing diabetes epidemic in Mexico.

**Supplementary Information:**

The online version contains supplementary material available at 10.1186/s12937-020-00642-9.

## Background

Type 2 diabetes is a major public health concern worldwide, and Mexico is not the exception [1]. Mexican national data indicate that there has been a marked increase in the prevalence of type 2 diabetes over the last two decades, from 6.7% in 1993 to 13.7% in 2016 [2, 3]. Since 2000, type 2 diabetes is the leading cause of death in Mexico, causing the majority of premature deaths [4]. A previous simulation study in Mexican population estimated that the prevalence of type 2 diabetes will follow a growing trend that could reach a prevalence of 22% in 2050 [5], leading to an increase in the demand for treatment that is unsustainable for any health system in low- and middle-income countries. In 2015 in Mexico, the total amount spent on health care for type 2 diabetes and its complications reached US$8.9 billion (US$3.9 billion in direct costs and US$5.0 in indirect costs) [6].

Diet is one of the leading risk factors for diabetes development [7], and sugar-sweetened beverages (SSBs) are among the dietary components that contribute most to the total energy intake in Mexican population [8]. Globally, Mexico is the country with the greatest mortality due to the consumption of SSBs, estimated to be around 405 per million adults for 2015, whereas one of every six diabetes-related disability-adjusted life years (DALYs) are attributable to these drinks [9].

Several studies have reported a positive association between the regular consumption of SSBs and type 2 diabetes risk [10, 11]; however, the existing research has almost exclusively focused on high-income countries. The large burden of disease related to type 2 diabetes in Mexico and the common exposure to soft drinks among our population are irrefutable. Nevertheless, there is scarce evidence to quantify the magnitude of the association between soft drinks and type 2 diabetes risk in the Mexican population. A recently published study explored the risk of diabetes by soda consumption in Mexican adults. However, this study was conducted only in women, and diabetes was self-reported [12]. Our study addresses the need to estimate the risk of type 2 diabetes (defined using three different criteria) due to soft drinks consumption among Mexican population. Country-specific estimates of type 2 diabetes risk are necessary for more precisely predicting the impact of potential interventions and aid to prioritize and planning actions with the greatest potential for success.

Furthermore, it is well known that certain ethnic populations, such as the Latin American population, have biological and phenotypical characteristics that predispose them to a higher risk of type 2 diabetes [13]. Therefore, we considered relevant to understand if the inherent excess risk in individuals with family history of diabetes can modify the association between soft drinks and risk of type 2 diabetes. We hypothesized that having a family history of diabetes could have a synergistic effect with soft drinks that could increase the type 2 diabetes risk.

## Methods

### Study design and participants

This study is a longitudinal analysis of the Health Workers Cohort Study (HWCS), an ongoing prospective cohort study established in January 2004 with two cohort follow-up waves at six-year intervals on average. The participants are employees from three different health and academic institutions, as well as their relatives, from the cities of Cuernavaca and Toluca, Mexico.

### Study population

**Fig. 1:**
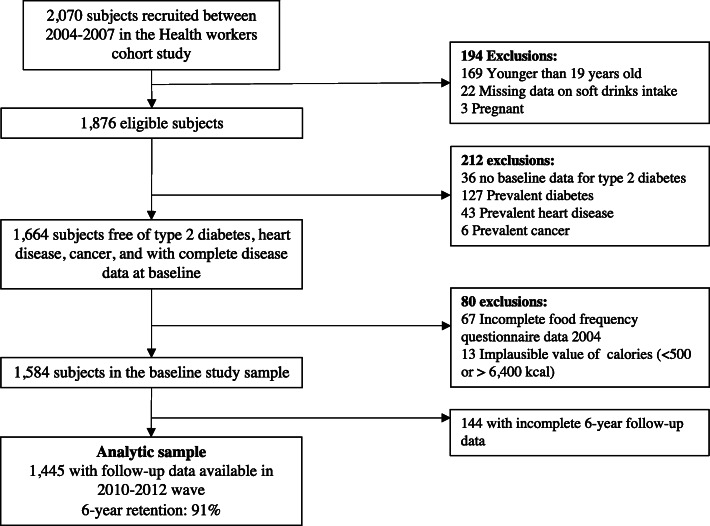
Flow chart of the study participants from the Health Workers Cohort Study included in the analytic sample, from 2004 to 2013

Details concerning the study population of HWCS and full study design have been described elsewhere [14]. Briefly, from January 7, 2004 to November 27, 2007, 10,729 participants aged 6–94 years old, were recruited. However, due to financial constraints, only 2500 (23.3%) of the initially enrolled participants from Cuernavaca were invited to the first follow-up phase between 2010 and 2013, with a response rate of 83% (*n* = 2070). Figure 1 shows the flow chart of the included participants in this study. For our analysis, we excluded participants who at baseline were younger than 19 years old (*n* = 169), who had missing data on soft drinks (*n* = 22), as well as pregnant women at baseline (*n* = 3). Subjects with missing type 2 diabetes baseline data (*n* = 36) or with previously known or newly diagnosed diabetes (*n* = 127), heart disease (*n* = 43), or cancer (*n* = 6) (except skin or melanoma) at baseline, were excluded. We also excluded 80 participants who responded < 75% of the food-frequency questionnaires (FFQs), had missing data in an entire section of the FFQ or implausible energy consumption defined as those who were below a predefined limit of 500 kcal/d or above 6400 kcal/d, following the standard deviation method [15], as previously used in studies from this cohort [16, 17]. After excluding 144 participants with incomplete data for disease outcome at six-year follow-up, 1445 participants were used as our analytic sample.

### Soft drinks intake

Soft drinks consumption was assessed at baseline and the subsequent examinations with a semi-quantitative 116-item FFQ that has been validated in the Mexican population [18]. Participants were asked to report the frequency of consumption of a standard portion of each food in the last 12 months using ten possible responses (never, < 1 time/month, 1–3/month, 1, 2–4, 5–6 times/week, 1, 2–3, 4–5, 6 or more times/d). Soft drinks were defined as cola soft drinks and flavored carbonated soft drinks with a standard serving of 355 ml. We converted the reported frequency of soft drinks into a daily intake. The frequency was converted into four categories of intake (< 1/month, 1–4,/month, 2–6/week, and ≥ 1/d) to get comparable data of soft drinks consumption with previous studies [19]. However, due to most participants were in the middle two categories of consumption (74.1%), we reclassified the categories of exposure as follows: < 1 time/week, 1–4 times/week, and > 5 times/week.

### Type 2 diabetes

Incident type 2 diabetes was defined as having one of the following three criteria during follow-up: self-report of physician-diagnosed type 2 diabetes, new use of hypoglycemic medication, or fasting glucose > 126 mg/dL during the examination [20]. A fasting venous blood sample (fasting time ≥ 8 h) was collected from each participant. We measured fasting glucose with the enzymatic colorimetric method by using glucose oxidize with a Selectra XL instrument (Randox, ELITechGroup, Delhi, India). The onset of type 2 diabetes was defined based on either the date of the follow-up examination or the year of physician diagnosis self-reported by the participants. Intervals of one-year between the two examinations were included in the questionnaire to record the time since type 2 diabetes diagnosis. June 30th was set as the diagnosis date for each year. We estimated the date of physician diagnosis subtracting the date of type 2 diabetes diagnosis to the date when completed questionnaires were returned.

### Covariates

At each study wave, participants completed a self-administered questionnaire that included information regarding demographic characteristics (age, sex, and educational level), previous and current illnesses, family history of diabetes, medication use, and lifestyle habits (smoking status and physical activity). We used the same measurement instruments for time-varying covariates to ensure comparability across waves. Educational level was categorized as middle school or less, high school, college or more. Participants were classified according to smoking status as never, former, and current smokers. Alcohol consumption (in g/d) was estimated from FFQ and categorized in tertiles. We calculated total energy intakes in kilocalories by multiplying the frequency of consumption of each food by the energy content of the food and summing over all foods. Leisure time of physical activity was assessed through a validated physical activity questionnaire [21]. Participants were asked to report the weekly leisure time to 16 activity items like walking, running, and cycling. Participants were classified as active if their leisure time of physical activity was ≥150 min/week [22].

Medical examinations and anthropometric measurements were also performed. All anthropometric measurements were performed by nurses trained to use standardized procedures. Reproducibility was evaluated, resulting in concordance coefficients between 0.83 and 0.90. Weight was assessed on participants wearing minimal clothing with a previously calibrated electronic TANITA scale. Height was measured with a conventional stadiometer. Body mass index (BMI) was calculated as weight (kg) divided by the square of height (m^2^). Waist circumference (WC) was measured midway between the lowest border of the rib cage and the upper border of the iliac crest, while the participant was standing up. We defined abdominal obesity as waist circumference > 90 cm for men and > 80 cm for women [23]. Resting blood pressure (mmHg) was measured twice using an automatic digital blood pressure monitor, and the average of two measurements was calculated. Subjects with a systolic or diastolic blood pressure of > 140 mmHg or > 90 mmHg, respectively, as well as those who reported use of antihypertensive medication, were classified as hypertensive.

### Statistical analysis

The study sample characteristics across categories of soft-drinks intake were described as means and standard deviation, as medians with interquartile ranges (IQR) for skewed distributions, or percentages for categorical variables. Because of the frequency of missing data at baseline for smoking status (3.5%), education level (2.4%), and abdominal obesity (1.4%), we used a missing indicator category for these covariates to minimize sample size reduction. We calculated person-years of follow-up from the date of returning the baseline questionnaire to the date of type 2 diabetes diagnosis or were censored on the date of their final follow-up visit. To examine the association of soft drinks consumption at baseline with type 2 diabetes, hazard ratios (HRs) along with 95% confidence intervals (CIs) were estimated using Cox proportional hazards regression with the time on study as the time scale. The category of < 1 time/week was considered as the reference group in all analyses.

Several models were fitted to assess the relationship between soft drinks intake and type 2 diabetes incidence. Model 1 was adjusted only for the age of participants (centered, continuous variable). Model 2 was further adjusted for potential confounders identified after reviewing the literature and by using the causal diagram methodology to select all variables related to the exposure and outcome. We considered the following covariates in the multivariate-adjusted analyses: sex, educational level (middle school or less, high school, college or more, missing), total energy intake (continuous), smoking status (never, former, current, missing), leisure-time physical activity in hours per week (active ≥150 min/week), family history of diabetes (no, yes, unknown), and alcohol intake at baseline (tertiles of g/d). Multivariable model 2 was further adjusted for hypertension status (no/yes), to test the potential confounding effect of hypertension in the association of soft drinks and type 2 diabetes. Some studies have suggested that having hypertension increases the risk of type 2 diabetes, while at the same time assuming that hypertensive individuals can alter their soft drinks consumption [24, 25]. To examine the potential confounding effect of obesity, we additionally adjusted model 2 for BMI and abdominal obesity at baseline, separately. People with overweight and obesity are more likely to have more energy-dense diets, including soft drinks, than people with healthy weight [26]. On the other hand, obesity is a leading risk factor for type 2 diabetes [27].

The potential modifying effect of first-degree family history of diabetes, as a proxy for genetic susceptibility for type 2 diabetes risk [13, 28], was evaluated by stratification on the family history of diabetes, and HRs within each stratum were compared. Also, we examined the overall interaction using the Wald test. This analysis just included the information of participants who responded yes or no in the variable of family history of diabetes (*n* = 1339). We conducted tests for a linear trend in the HRs by assigning the median value to each category of soft drinks and modeling this variable as a continuous variable into separate Cox regression models (adjusting by the same covariates). The proportional hazards assumption was assessed by a graphical check on the cumulative log hazard versus time and tested by using Schoenfeld residuals [29], which test the null hypothesis of zero slopes for individual covariates and globally for each regression model. The assumption of proportional hazards was not violated (*P* > 0.05).

### Sensitivity analysis

We conducted a complete case analysis using data from those participants with complete follow-up data from 2004 to 2018 (*n* = 600). The complete case approach was not considered as main analysis because the smaller sample size and large loss to follow-up that could affect estimates through selection bias. We are aware that potential changes in soft drink consumption over time due to the ageing of the cohort may impact the soft drink consumption [30]. For this purpose, we further used Cox proportional hazards models where the soft drinks consumption was updated from the follow-up questionnaire and considered as a time-varying variable in these regressions.

All *P*-values were two-tailed and *P* < 0.05 was considered significant. Statistical analysis was performed using Stata version 14.0 (StataCorp, College Station, TX, USA).

## Results

### Baseline characteristics

Our analytic sample comprised 1445 participants with a mean of age of 44.3 ± 12.5 years, most of them women (75.6%). Mean BMI in all participants was 26.2 ± 4.1 kg/m^2^, and more than half of them (58.4%) were overweight or obese at baseline. The median intake of soft drinks among our participants was 0.2 servings/day (IQR 0.10–0.57). Half of the sample consumed 1–4 servings per week at baseline, whereas 21.7% consumed > 5 servings per week.

Table 1 shows the characteristics of the participants at baseline across levels of soft drinks consumption. The participants in the highest level of soft drinks consumption (≥5/week) had more obesity, were less physically active, had higher fasting glucose levels, and higher total energy intake than individuals consuming < 5/week. Participants in the top level of soft drinks consumption tend to be more alcohol drinkers and current smokers.

**Table 1:** Characteristics of participants in the HWCS^a^ according to their soft drinks’ intake at baseline 2004–2006 (*n* = 1445)

Characteristics	Consumption level		
	< 1 per week	1–4 per week	> 5 per week
	*n* = 361 (25.0%)	*n* = 770 (53.3%)	*n* = 314 (21.7%)
Sex, %			
Women	88.4	76.6	58.3
Men	11.6	23.4	41.7
Age (years)^b^	46.4 ± 12.5	43.9 ± 12.6	42.8 ± 12.0
Education, %			
Middle school or less	24.4	25.6	27.4
High school	16.6	25.6	25.2
≥ College	55.4	46.6	45.9
Missing	3.6	2.2	1.6
Weight (kg)^c^	61.3 (55.6–68.6)	65.0 (58.1–73.1)	68.7 (58.8–78.0)
Body mass index (kg/m^2^)^b^	25.5 ± 4.0	26.4 ± 4.0	26.7 ± 4.5
Body mass index categories, %			
Normal	52.8	37.6	38.2
Overweight	35.3	45.1	40.5
Obesity	11.9	17.3	21.3
Waist circumference (cm)^c^	85 (79–94)	89 (82–97)	91 (84–99)
Abdominal obesity, %	68.7	73.8	73.2
Missing	1.4	1.2	1.9
Leisure-time physical activity (hrs. Per week)^b^	1.5 (0.4–4.6)	1.5 (0.4–3.9)	1.5 (0.2–4.3)
Active (≥ 150 min/week), %	42.7	37.8	35.1
Family history of diabetes, %			
No	40.8	39.9	42.3
Yes	52.5	52.6	53.2
Unknown	6.7	7.6	4.5
Fasting glucose (mg/dL)^b^	88.2 ± 9.3	89.7 ± 10.4	91.2 ± 11.6
Hypertension, %	16.9	15.9	17.1
Total energy intake (kcal/d)^b^	2008 ± 862	2100 ± 835	2437 ± 903
Smoking, %			
Never	62.3	55.6	44.9
Former	22.2	24.3	26.8
Current	10.8	16.6	27.4
Missing	4.7	3.5	1.9
Alcohol intake (g/d)^c^	0.8 (0.04–2.0)	1.0 (0.3–4.0)	1.7 (0.6–6.8)
Alcohol intake categories, %			
Tertile 1 (< 0.6 g/d)	45.7	32.3	26.1
Tertile 2 (0.6–2.4 g/d)	33.0	34.2	29.6
Tertile 3 (> 2.4 g/d)	21.3	33.5	44.3

^a^HWCS, Health Workers Cohort Study, ^b^Mean ± standard deviation, ^c^p50 (interquartile range)

### Soft drinks and type 2 diabetes incidence

Table 2 presents the risk of diabetes according to categories of soft drinks intake. During 9526 person-years of follow-up with a median follow-up 6.7 years (interquartile range: 6.2–7.1 years) in 1445 normoglycaemic individuals at baseline, we ascertained 109 incident cases of type 2 diabetes, yielding an incidence rate of 11.4 per 1000 person-years (95%CI 9.4–13.8) in the whole study population. Type 2 diabetes risk increased with increasing intake of soft drinks (*p* < 0.001 for trend). The crude incidence rate of type 2 diabetes was 7.6, 11.0, and 17.1 per 1000 person-years across levels of soft drinks consumption of < 1/week, 1–4 /week, and > 5/week, respectively. After adjustment for age, sex, educational level, total energy intake, physical activity, smoking status, family history of diabetes, and alcohol intake (model 2) the risk of type 2 diabetes among individuals with soft drinks consumption ≥5/week was two-fold higher (HR 1.9; 95% CI, 1.0–3.5) compared with those in the lowest level of consumption (< 1/week) (p-trend = 0.040). The hazard ratios for type 2 diabetes across the levels of soft drinks consumption remained similar after adjustment for hypertension status, while the hazard ratios were attenuated when models were adjusted for BMI (HR 1.5, 95%CI: 0.8–2.8) or abdominal obesity (HR 1.6, 95%CI: 0.8–3.0).

**Table 2:** Risk of Type 2 Diabetes according to categories of soft drinks intake in participants from HWCS (*n* = 1445)

	Consumption level at baseline			
	< 1/week	1–4 /week	> 5/week	*p*_trend_^a^
Median (IQR), servings per week	0.2 (0.1–0.2)	1.5 (1.1–3.1)	7.1 (6.0–10.0)	< 0.001
n	361	770	314	
Cases of type 2 diabetes (*n* = 109)	18	56	35	
Person-years	2371.8	5113.4	2040.9	
Crude incidence rate (per 1000)	7.6 (4.8–12.0)	11.0 (8.4–14.2)	17.1 (12.3–23.8)	
Model 1 - Age-adjusted, HR (95% CI)	Ref.	1.5 (0.9–2.5)	2.3 (1.3–4.0)	0.004
Model 2 - Multivariate-adjusted^b^	Ref.	1.3 (0.7–2.2)	1.9 (1.0–3.5)	0.040
Model 3 - Model 2 + hypertension, HR (95% CI)	Ref.	1.3 (0.7–2.2)	1.8 (1.0–3.3)	0.046
Model 4 - Model 2 + baseline BMI, HR (95% CI)	Ref.	1.0 (0.6–1.7)	1.5 (0.8–2.8)	0.094
Model 5 - Model 2 + abdominal obesity, HR (95% CI)	Ref.	1.1 (0.6–1.9)	1.6 (0.8–3.0)	0.083

*Abbreviations*: *HWCS* Health Workers Cohort Study, *IQR* Interquartile range, *HR* Hazard risk, *CI* Confidence interval, *BMI* Body mass index

^a^A linear trend in the HR for each of the soft drinks categories was evaluated by including a continuous variable in the model representing the median values of each of soft drinks intake

^b^Adjusted for baseline covariates: age centered, sex, total energy intake, physical activity, smoking status, family history of diabetes, and alcohol intake at baseline (tertiles), level of education

Results of our stratified analysis showed differences in the association between soft drinks and type diabetes across family history of diabetes, while not significantly different (*p* = 0.4285 for overall interaction) (Table 3). The subgroup of participants with family history of type 2 diabetes showed a statistically significant positive association comparing top and bottom soft drinks intake (HR = 2.3; CI: 1.04–5.17, p-trend = 0.037), whereas in those without family history there was a not significant risk reduction (HR = 0.6, p-trend = 0.674).

**Table 3:** Risk of Type 2 Diabetes according to categories of soft drinks intake in participants from HWCS stratified by family history of diabetes (yes/no)^a^

	Consumption level			
	< 1/week	1–4 /week	> 5/week	*p*_trend_^a^
Subjects without family history of diabetes (*n* = 582)				
n	147	303	132	
Cases of type 2 diabetes (*n* = 25)	7	12	6	
Person-years	969.4	2032.2	862.6	
Incidence rate (per 1000)	7.2 (3.4–15.1)	5.9 (3.4–10.4)	7.0 (3.1–15.5)	
Multivariate-adjusted^2^, HR (95% CI)^b^	Ref.	0.67 (0.25–1.79)	0.66 (0.20–2.16)	0.674
Subjects with family history of diabetes (*n* = 757)				
n	189	402	166	
Cases of type 2 diabetes (*n* = 74)	10	38	26	
Person-years	1233.4	2673.9	1078.5	
Incidence rate (per 1000)	8.1 (4.4–15.1)	14.2 (10.3–19.5)	24.0 (16.1–35.4)	
Multivariate-adjusted, HR (95% CI)^b^	Ref.	1.49 (0.73–3.07)	2.3 (1.04–5.17)	0.037

*Abbreviations*: *HWCS* Health Workers Cohort Study, *IQR* Interquartile range, *HR* Hazard risk, *CI* Confidence interval

P for overall interaction = 0.4285

^a^ A linear trend in the HR for each of the soft drinks categories was evaluated by including a continuous variable in the model representing the median values of each of soft drinks intake

^b^ Adjusted for baseline covariates: age centered, sex, total energy intake, physical activity, smoking status, alcohol intake at baseline (in tertiles) and level of education

### Result of sensitivity analyses

In the complete case data analysis, we included 600 subjects with a median follow-up of 12.5 years per subject. We observed that the participants included in the complete case analysis were older, had a larger waist circumference, and a lower proportion were current smokers than those with incomplete follow data (Additional File 1, Supplemental Table 1). Overall, there were 108 incident cases of type 2 diabetes during a total of 7081.6 person-years of follow-up. Participants with the highest consumption of drinks intake had a risk of type 2 diabetes 2.4 times (95% CI 1.3–4.6) the risk among those that consumed < 1 serving/week of soft drinks. The complete case analysis when soft drinks consumption was considered as a time-varying variable gave comparable results as our main analysis. (Additional File 1, Supplemental Table 2).

## Discussion

We aimed to estimate the risk of type 2 diabetes associated with soft drinks consumption in a cohort of Mexican adults. After 6.7 years of follow-up, we observed that participants who consumed five or more servings of soft drinks per week at baseline experienced twice the risk of type 2 diabetes, compared to participants who consumed less than one serving per week. This association was robust to multivariate adjustment and complete case analysis but attenuated when models were further adjusted for BMI or abdominal obesity.

The link between SSBs and type 2 diabetes has been well established in longitudinal studies from high-income countries in Europe and North America. Primarily based on those studies, Imamura et al., estimated that diabetes risk increased 18% for every SSBs serving consumed per day [10]. Malik et al., in their meta-analysis, estimated that people who consumed one or more servings of SSBs per day had 26% higher risk for developing diabetes, compared to individuals who consumed less than one serving/month [11]. These two estimates are aligned with our estimate of twice the risk of diabetes when people consume five servings per day or more, suggesting that there is no heterogeneity of effects for the Mexican population than the populations included in those meta-analyses. This risk level is worrisome, considering that more than 20% of our cohort of health workers and their relatives reported drinking five or more servings of soft drinks per week at baseline. In the 2016 National Health and Nutrition Survey, an estimated 80% of the adult population reported consuming sweetened beverages ≥3/week [31], suggesting that soft drinks are a primary risk factor to be reduced to prevent diabetes cases.

The finding of a higher risk of type 2 diabetes among adults in the highest versus the lowest category of soft drinks intake is consistent with a previous study using data from the Mexican Teachers’ Cohort (MTC) [12]. Whereas the hazard ratio in individuals who consumed five or more servings per week in our study is higher than the observed in the MTC (HR 1.9 vs. 1.3), the difference in the categories used as reference does not allow a direct comparison between the estimators. The reference was < 1 serving/week and one serving/day in HWCS and MTC studies, respectively. Additionally, there were some important differences in the methods between our study and the MTC, including different definitions of type 2 diabetes and the longer time to follow-up in our study (6.7 years in the HWCS versus 2.2 years in MTC), which could explain the HRs found. Despite the heterogeneity of the populations studied, both studies agree that consuming one serving or more per day of soft drinks increases type 2 diabetes risk.

Several biological mechanisms have been proposed to explain the positive association between SSBs and the risk of type 2 diabetes, yet, two main pathways are widely recognized in the literature. First, the glycemic effect of beverages on insulin demand can result in glucose intolerance and insulin resistance [32, 33]. Second, SSBs also increase the risk of diabetes through weight gain, which occurs because of a caloric surplus that is not compensated [34]. Finally, recent studies suggest that high fructose corn syrup, a common sweetener used in soft drinks, tends to increase visceral adiposity, which is associated with insulin resistance and other metabolic complications [35].

We also tested the hypothesis that participants with a family history of diabetes would be more susceptible to develop type 2 diabetes when exposed to soft drinks. There is evidence that first-degree relatives of patients with diabetes have a 30–70% increased risk of developing the disease [28]. However, very few studies have addressed this question. The previously mentioned cohort study in Mexican women, documented that the interaction of family history of diabetes and soft-drinks was not statistically significant [12]. We found evidence for an association between soft drinks consumption and diabetes incidence within the subgroup of those having a family history of diabetes, but not for the contrary stratum. It is important to note that the number of events in the group with a family history of diabetes was three times greater than those reported not having. Using family history of diabetes as a proxy for genetic susceptibility is a limited approach, and perhaps the assessment of specific genes that have been linked to type 2 diabetes in Mexican mestizos could produce a different result [36]. Beyond the correct classification of genetic susceptibility, recent evidence suggests that epigenetic changes by regular consumption of SSBs may have negative consequences on adiposity in people with genetic predisposition to obesity [37]. Future genetic studies will be needed to understand better the potential epigenetic changes produced by soft drinks and their interactions in populations with greater genetic susceptibility to type 2 diabetes.

This study has limitations that we acknowledge. First, the data on diet consumption was collected using a FFQ, which has been shown to underestimate the true soft drinks consumption, given the perceived social stigma associated with these unhealthy beverages [38, 39]. However, differential exposure misclassification is unlikely due to the prospective design of the study. We acknowledge that the number of events in our group of participants without a family history of diabetes is inadequate to address comparisons across soft-drinks consumption categories. The width of the CIs suggests that our study was not powered to investigate such differences. Therefore, the results should be interpreted with caution. Also, we are aware that the association between soft drinks and type 2 diabetes risk can be mediated by weight gain [40]. However, we did not conduct a mediation analysis since the three-wave sample’s power is insufficient to detect a change in the association’s magnitude, assuming a proportion mediated by obesity of 23% [26]. Indeed, we just reach a power of 48% to estimate an attenuation of our HR from 1.9 to 1.5. Future cohort studies with multiple-repeated measurements and larger samples can help to address mediation analysis properly. Another limitation was the high attrition rate in our cohort that could induce selection bias. Our sensitivity analysis found that the estimates for the complete case analysis using baseline soft drinks consumption were relatively higher than those obtained from the main analysis. It is then likely that data were not missing at random; by providing our estimates based on the main analysis, we expect to attenuate the potential selection bias that could occur if based on a complete-case analysis. Finally, the representativeness was not a concern of our study since the overall goal is to advance our understanding of the causal mechanisms towards type 2 diabetes. In this sense, though the individuals that compose our sample are just from an urban area of Cuernavaca, Mexico, who had a higher level of schooling than the national average, there is no biological rationale to expect that the association found varies by education level or region. It is worth noting that the prevalence of 6.9% of type 2 diabetes found at baseline in our study (2004–2006) is very similar to the nationwide prevalence of 7.0% reported in the Mexican National Health and Nutrition Survey in 2006 [41].

Despite the limitations, our study also has several strengths. First, diabetes was determined by using three different criteria, including fasting glucose, reducing the potential disease misclassification. Second, the median age of our study population and the long follow-up period gave us an appropriate time frame to observe an important number of new diabetes cases. Finally, this is the first study, to our knowledge, examining the association between soft drinks and risk of type 2 diabetes in both Mexican men and women.

## Conclusion

Our results are valuable to support the recommendations that reducing soft drinks intake is a key target to address preventive interventions to reduce chronic diseases as type 2 diabetes incidence and its complications. Providing local evidence about the risk of type 2 diabetes associated with regular consumption of soft drinks is vital for predicting the impact of potential interventions and leading to better allocation of resources, especially considering the growing epidemic of diabetes in low- and middle-income countries.

## Supplementary Information

**Additional file 1:** **Supplemental Table 1.** Baseline characteristics of the participants from the case complete analysis as compared with those who lost to follow-up either in any of the follow-up waves of the cohort^1^. **Supplemental Table 2.** Risk of Type 2 Diabetes by soft drinks consumption in participants from HWCS^1^ who complied with the entire follow-ups at wave-2 (2010–2013) and wave-3 (2016–2018) (*n* = 600).

## Data Availability

The datasets used and/or analyzed during the current study are available from the corresponding author on reasonable request.

## Abbreviations

BMI: Body mass index
CI: Confidence intervals
d: Day
DALYs: Disability-adjusted life years
FFQ: Food-frequency questionnaire
HRs: Hazard ratios
HWCS: Health workers cohort study
IQR: Interquartile ranges
kcal: Kilocalorie
MTC: Mexican teachers’ cohort
mmhg: Millimeter of mercury (mmHg)
SSBs: Sugar-sweetened beverages
WC: Waist circumference
