# Supplementary material for: Regular consumption of soft drinks is associated with type 2 diabetes incidence in Mexican adults: findings from a prospective cohort study
Source: Nutr J. 2020 Nov 20;19:126. doi: 10.1186/s12937-020-00642-9 (PMC7678283; doi:10.1186/s12937-020-00642-9)
Supplement: Supplementary file 1 — Additional file 1: Supplemental Table 1. Baseline characteristics of the participants from the case complete analysis as compared with those who lost to follow-up either in any of the follow-up waves of the cohort1. Supplemental Table 2. Risk of Type 2 Diabetes by soft drinks consumption in participants from HWCS1 who complied with the entire follow-ups at wave-2 (2010–2013) and wave-3 (2016–2018) (n = 600). [file 12937_2020_642_MOESM1_ESM.docx]

**Additional file 1**

| **Supplemental Table 1.** Baseline characteristics of the participants from the case complete analysis as compared with those who lost to follow-up either in any of the follow-up waves of the cohort^1^ | | | | |
| --- | --- | --- | --- | --- |
|  | **Baseline (people with complete data for 3waves)** | | |  |
| Characteristics | Complete follow-up n=600 (38%) | Lost to follow-up  n=984 (62%) | P-value |  |
| Women, % | 76.3 | 74.9 | 0.52 |  |
| Age (years) | 46 (38-55) | 42 (33-51) | <0.001 |  |
| Weight (kg) | 64.1 (57-73.2) | 64.6 (57.4-73) | 0.699 |  |
| Body mass index (kg/m^2^) | 25.8 (23.3-28.8) | 25.6 (23.5-28.4) | 0.556 |  |
| Body mass index categories, % |  |  |  |  |
| Normal | 41.1 | 42.6 | 0.545 |  |
| Overweight | 41.1 | 41.3 | 0.969 |  |
| Obesity | 17.5 | 16.1 | 0.449 |  |
| Waist circumference (cm) | 90 (83-99) | 88 (80-96) | <0.001 |  |
| Abdominal obesity, % | 78.0 | 70.2 | 0.001 |  |
| Leisure-time physical activity (hrs. per week) | 1.7 (0.38-4.63) | 1.5 (0.38-4.25) | 0.152 |  |
| Active (≥ 150 min/week), % | 41.5 | 37.6 | 0.123 |  |
| Family history of diabetes, % |  |  |  |  |
| No | 42 | 38.9 | 0.224 |  |
| Yes | 52.7 | 53.6 | 0.722 |  |
| Unknown | 5.3 | 7.5 | 0.095 |  |
| Fasting glucose (mg/dL) | 89 (82-95) | 89 (82-96) | 0.782 |  |
| Hypertension, % | 15.5 | 16.7 | 0.529 |  |
| Smoking, % |  |  |  |  |
| Never | 57.3 | 52.5 | 0.063 |  |
| Former | 24.5 | 24.9 | 0.858 |  |
| Current | 14.7 | 19.2 | 0.021 |  |
| Missing | 3.5 | 3.4 | 0.873 |  |
| Alcohol intake categories, % |  |  |  |  |
| Tertile 1 (<0.6 g/day) | 33.3 | 35.2 | 0.457 |  |
| Tertile 2 (0.6-2.4 g/day) | 31 | 34.5 | 0.157 |  |
| Tertile 3 (>2.4 g/day) | 35.7 | 30.4 | 0.029 |  |
| Total energy intake (kcal/day) | 1945.9 (1545-2584.4) | 2030.3 (1536.4-2575.2) | 0.543 |  |
| Soft drinks (servings/week) | 1.5 (0.7-4.0) | 1.5 (0.6-4.0) | 0.360 |  |

^1^ Values are median and interquartile range, unless otherwise indicated.

| **Supplemental Table 2.** Risk of Type 2 Diabetes by soft drinks consumption in participants from HWCS^1^ who complied with the entire follow-ups at wave-2 (2010-2013) and wave-3 (2016-2018) (n=600) | | | | | | | | |
| --- | --- | --- | --- | --- | --- | --- | --- | --- |
| **A)** |  | **Consumption level at baseline** | | | | | | |
|  |  | < 1/week |  | 1-4 /week |  | >5/week |  | *p* _trend_^1^ |
| Median (IQR), servings per week |  | 0.2 (0.1-0.2) |  | 1.5 (1.1-3.1) |  | 7.1 (6.0-8.6) |  | <0.001 |
| Cases of type 2 diabetes (n=108) |  | 16 |  | 56 |  | 36 |  |  |
| Person-years |  | 1687.2 |  | 3718.0 |  | 1676.4 |  |  |
| Incidence rate (per 1,000) |  | 9.5 (5.8-15.5) |  | 15.1 (11.6.19.6) |  | 21.5 (15.5-29.8) |  |  |
| Model 1 - Age-adjusted, HR (95% CI) |  | Ref. |  | 1.7 (1.0-3.1) |  | 2.9 (1.6-5.3) |  | <0.001 |
| Model 2 - Multivariate-adjusted,^3^ HR (95% CI) |  | Ref. |  | 1.6 (0.9-3.0) |  | 2.4 (1.3-4.6) |  | 0.011 |
|  |  |  |  |  |  |  |  |  |
| **B)** |  | **Consumption level as time varying^4^** | | | | | | |
|  |  | < 1/week |  | 1-4 /week |  | >5/week |  | *p* _trend_^1^ |
| Median (IQR), servings per week |  | 0.2 (0.1-0.2) |  | 1.1 (0.7-3.1) |  | 6.0 (3.0-7.6) |  | <0.001 |
| Cases of type 2 Diabetes (n=108) |  | 28 |  | 49 |  | 31 |  |  |
| Person-years |  | 1999.0 |  | 3528.0 |  | 1511.2 |  |  |
| Incidence rate (per 1000) |  | 14.0 (9.7-20.3) |  | 13.9 (10.5-18.4) |  | 20.5 (14.4-29.2) |  |  |
| Model 1- Age adjusted, HR (95% CI) |  | Ref. |  | 1.3 (0.8-2.1) |  | 2.3 (1.4-3.9) |  | 0.001 |
| Model 2 - Multivariate-adjusted,^3^ HR (95% CI) |  | Ref. |  | 1.2 (0.7-2.0) |  | 1.9 (1.1-3.4) |  | 0.020 |
| ^1^ HWCS, Health Workers Cohort Study; IQR; interquartile range; HR, hazard risk; CI, confidence interval; BMI, body mass index in categories (normal, overweight and obese).  ^2^A linear trend in the HR for each of the soft drinks categories was evaluated by including a continuous variable in the model representing the median values of each of soft drinks intake.  ^3^Adjusted for baseline covariates: age continuous (centered), sex, total energy intake, physical activity, smoking status, family history of diabetes, alcohol intake at baseline (in tertiles), and level of education.  ^4^ Soft drinks consumption was updated from the follow-up questionnaire (2^nd^ wave) and considered as time varying variable in the Cox models. | | | | | | | | |
